# Integrated transcriptomic and clinical prediction model for malignant pulmonary nodules: Model development, external dataset evaluation, and single-center expression validation

**DOI:** 10.1097/MD.0000000000051383

**Published:** 2026-10-30

**Authors:** Xingshu Zhang, Jie Yang, Ming Gong, Qian Luo, Yongxiang Song

**Affiliations:** a Department of Thoracic Surgery, Affiliated Hospital of Zunyi Medical University, Zunyi, China; b Department of Thoracic Surgery, First Affiliated Hospital of Guizhou University of Traditional Medicine, Guiyang, China.

**Keywords:** logistic regression, lung cancer, prediction model, pulmonary nodule, whole blood gene expression

## Abstract

Discriminating between benign and malignant pulmonary nodules remains a clinical challenge, complicating treatment decisions. This study developed a predictive model integrating transcriptomic and clinical data to characterize pulmonary nodules. Gene expression profiles and clinical data from GSE108375 were analyzed. Differential expression and weighted gene co-expression network analysis (WGCNA) were performed. Patients were randomly split into training and validation sets (7:3). The model was further evaluated using an external dataset (GSE225787), and candidate gene expression was examined in a single-center clinical cohort (n = 30) using blood reverse‑transcription quantitative real‑time PCR and nodule-tissue immunohistochemistry (IHC). WGCNA identified the MEgreen module as significantly associated with malignant nodules. Among 776 differentially expressed genes between malignant and benign nodules, 36 overlapped with the MEgreen module. Last absolute shrinkage and selection operator selected age, lesion size, and 5 genes (RBX1, GZMK, AKR1C3, NDUFA4, CELA1) for model construction. The model achieved AUCs of 85.16% in the training set and 80.23% in the internal validation set, with an AUC of 0.867 in the external dataset. In the single-center clinical cohort, blood CELA1 and NDUFA4 mRNA levels were significantly higher in the malignant group, and tissue IHC measurements of the corresponding proteins also differed significantly between groups. The integrated transcriptomic and clinical model showed potential for estimating malignancy risk in pulmonary nodules. The expression findings for CELA1 and NDUFA4 support further investigation of these candidate markers in larger clinical cohorts.

## 1. Introduction

Incidental pulmonary nodules are frequently identified on chest computed tomography (CT),^[1,2]^ however, their malignant potential is often unclear at initial detection. False-positive CT findings may lead to additional radiation exposure, invasive biopsy-related complications, and patient anxiety.^[3,4]^ Accurate discrimination between benign and malignant pulmonary nodules is therefore of considerable clinical importance.^[5,6]^ Previous studies have indicated that predictors such as size, growth rate (volume doubling time < 400 days), age, smoking, and extrapulmonary tumor history have the potential to predict the malignancy of pulmonary nodules.^[7,8]^ Clinical prediction models, including the Mayo Clinic and Veterans Affairs (VA) models, have also been widely used in clinical practice. However, due to the absence of specific lung cancer biomarkers, current tools still lack sufficient accuracy to reliably classify pulmonary nodules and guide subsequent management.^[9,10]^

Beyond radiological assessment, several blood-based molecular classifiers have been developed to enhance diagnostic precision. For instance, Kossenkov et al^[6]^ demonstrated that a gene expression classifier derived from peripheral blood RNA could effectively distinguish malignant from benign nodules. Other proteomic-based^[11]^ and DNA methylation-based models^[12]^ have also shown promise. Furthermore, inflammatory markers^[13]^ and large-scale clinical cohorts^[14]^ have been utilized to construct predictive frameworks. Despite these advancements, there remains a need for an integrated model that combines specific transcriptomic signatures with clinical characteristics, supported by multi-level experimental validation.

Although circulating tumor cells (CTCs) offer diagnostic and prognostic biomarkers, they are generally unsuitable for cancer screening due to the prolonged time required for antibody-based recognition and enrichment. In contrast, peripheral blood mononuclear cells (PBMCs) represent a viable alternative. Blood gene expression offers a potential source of minimally invasive diagnostic markers. Previous studies have established a foundation for the use of PBMCs in cancer diagnostics. For instance, Showe et al demonstrated that PBMC gene expression profiles could differentiate patients with early-stage non-small cell lung cancer (NSCLC) from non-cancer controls, suggesting that PBMCs reflect the systemic response to cancer presence.^[15]^ These findings support the investigation of circulating-cell transcriptomes as a source of diagnostic markers. Furthermore, a patent by Zhou et al described methods for diagnosing lung cancer using PBMC-derived gene expression signatures, underscoring the potential of these cells as biomarkers for early detection.^[16]^ In this study, we developed a prediction model combining whole-blood mRNA expression profiles with clinical characteristics of patients with pulmonary nodules from GEO and examined candidate marker expression in a single-center clinical cohort.

## 2. Materials and methods

### 2.1. Data acquisition and processing

The GSE108375 dataset was downloaded from GEO datasets (https://www.ncbi.nlm.nih.gov/gds/). This dataset was produced by using the GPL10558 Illumina HumanHT-12 V4.0 expression beadchip and contained 151 samples of benign nodules (BNs) and 164 samples of malignant nodules (MNs). GSES6225787 is a sequencing dataset of whole blood from 30 lung cancer patients and 27 normal control patients. We use GSE225787 to perform external validation of the model.

### 2.2. WGCNA analysis

We used the weighted gene co-expression network analysis (WGCNA) algorithm to identify coexpressed genes and coexpression modules. The Pearson correlation coefficient was used to calculate the distance between each gene, and a weighted gene coexpression network was constructed by the WGCNA package (version 1.71). The pickSoftThreshold function was used to determine the soft-thresholding power, with a scale-free topology fit index (*R*^2^) ≥ 0.8 set as the criterion for selecting an appropriate soft threshold. The gene expression matrix was subsequently transformed into an adjacency matrix and then into a topological overlap matrix (TOM). Co-expression modules were identified using the blockwiseModules function, with minModuleSize = 30 and mergeCutHeight = 0.25. After excluding unassigned genes, we visualized the resulting modules via a TOM heatmap.

### 2.3. Differential expression and enrichment analysis

Differentially expressed genes between MNs and BNs were identified using the “limma” R package, with |logFC| > [mean(|logFC|) + 2 sd(|logFC|)] as the standard for significance differences. Gene ontology (GO) enrichment and Kyoto Encyclopedia of Genes and Genomes (KEGG) pathway analysis were conducted using the clusterProfiler R package (4.0). Biological processes (BP), cellular components (CC), and molecular function (MF) were included in the GO enrichment analysis. The online search tool (STRING, version 11.0) database was utilized to map common genes and assess their interactive relationships. Interactions with a combined score >0.4 were included in the network. Metascape analysis was performed through the Metascape website (https://Metascape.org/). Gene set variation analysis (GSVA) was applied as a nonparametric unsupervised method to evaluate the enrichment of gene sets derived from the Molecular Signatures Database (v7.0). Each set was scored using the GSVA algorithm, and potential differences in signaling pathways between the MN and BN groups were examined. Spearman correlation analysis was then conducted between the gene set and the predicted sample score.

### 2.4. Data preprocessing and batch correction

Raw gene expression profiles from all cohorts were first background-corrected and log2‐transformed. For microarray datasets, the Robust Multiarray Average (RMA) algorithm was applied using the affy package (Bioconductor v1.72.0); RNA-seq counts were normalized by the trimmed mean of *M*-values (TMM) method via edgeR (v3.36.0). All expression matrices were then quantile-normalized to ensure comparability across platforms. Batch effects arising from different platforms and collection time points were assessed by principal component analysis and the surrogate variable analysis (SVA) package (v3.42.0). Identified surrogate variables were incorporated into a ComBat correction model (sva::ComBat) to adjust for batch differences. Post-correction PCA confirmed removal of platform-specific clustering, and adjusted data were used for all downstream differential expression and model-building steps.

### 2.5. Clinical prediction model construction

Utilizing the Glmnet package (R v4.1-4), last absolute shrinkage and selection operator (LASSO) was applied to whole-blood differentially expressed genes. The optimal penalty parameter λ was determined via cv.glmnet with 10-fold cross-validation, selecting both the minimum-error λ and the 1-SE λ; this procedure was repeated 3 times within an inner 5-fold CV loop to ensure feature stability. Selected genes were included in a multivariate logistic regression (significance threshold *P* < .05) to derive the diagnostic signature and construct a nomogram based on regression coefficients. Calibration of the nomogram was assessed using the calibrate function in the rms package (v5.1-4) with 1000 bootstrap resamples, plotting predicted probabilities against observed outcomes. Discriminative performance was evaluated by ROC analysis via the pROC package, reporting mean AUC and 95% CI across an outer 5-fold CV. Decision curve analysis using the dca package (v0.1.0.9) was used to evaluate potential net benefit across threshold probabilities.

### 2.6. A clinical cohort study constructed to validate the differential expression of model genes

To validate the differential expression of the model genes, we constructed a single-center cohort study comprising 30 patients who underwent pulmonary nodule resection in the Department of Thoracic Surgery at the Affiliated Hospital of Zunyi Medical University between August 25, 2022 and August 24, 2023. This study was approved by the Biomedical Research Ethics Committee of Zunyi Medical University Affiliated Hospital (No. KLL-2022-678). All participants provided written informed consent.

The inclusion criteria were as follows: Pulmonary nodules were discovered through chest thin-layer CT examination within 2 weeks prior to surgery; older than 18 years old, regardless of gender; without any treatment for pulmonary nodules before surgery; definitive pathological diagnosis was obtained for all nodules via surgical resection and histological examination; no mediastinal lymph node enlargement, atelectasis, or pleural effusion; normal cognitive ability, no diseases that affect cognition such as Alzheimer’s or schizophrenia; voluntarily participated in this study and signed an informed consent form.

Exclusion criteria were as follows: unclear imaging in thin-layer CT examination; pulmonary nodules diagnosed as metastatic lesion through pathological examination; pregnant and lactating women. After admission, 2 mL of venous blood was extracted from patients for RNA extraction and subsequent expression validation of the candidate genes via reverse‑transcription quantitative real‑time PCR (RT-qPCR).

### 2.7. RNA isolation, RT-qPCR

Total RNA was extracted from whole blood using the RNArep Pure High Efficiency Blood Total RNA Extraction Kit (TIANGEN Co., Ltd, China). Reverse transcription was conducted by PrimeScript™ RT Mix (Takara, China) to obtain cDNA. We performed QPCR analysis on a QuantStudio 7 Flex (Applied Biosystems). PCR conditions were 1 cycle of initial denaturation at 95°C for 3 minutes. After that, 40 cycles of denaturation with 10 seconds at 95°C, annealing and extension at 60°C for 30 seconds.^[17]^ Target gene expression was normalized to GAPDH within each sample and reported as relative expression. The primer sequence (see Table 1) was downloaded from Primer bank (https://pga.mgh.harvard.edu/primerbank/index.html) and synthesized by Shanghai Sangon Biotechnology (Shanghai, China). All experiments were performed in triplicate.

**Table 1:** The primers of genes measured using RT-qPCR.

Primers	Forward primer (5’ → 3’)	Reverse primer(5’ → 3’)
RBX1	GCTCAAGGCAGAGTGTGGAT	GCCATCAGTACCCAAGCTGT
GZMK	CTCCATCCAGTATGGCGGAC	GTGTGCGCCTAAAACCACAG
AKR1C3	TAGCCAGCTGAGTGACAGTGAT	AATCCCAGGACAGGCATGAAGTG
NDUFA4	TCTCTTGCGTCTGGCATTGT	TGGGACCCAGTTTGTTCCAG
CELA1	CAGTGGTCTACTCCATCGGC	CAGGTGTGATACCGGGAACC
PPIA	GTGGTGTTTGGCAAAGTGAA	TCGAGTTGTCCACAGTCAGC
GAPDH	GGAGCGAGATCCCTCCAAAAT	GGCTGTTGTCATACTTCTCATGG

RT-qPCR = reverse-transcription quantitative real-time PCR.

### 2.8. Immunohistochemistry

Resected pulmonary nodules were fixed in 10% paraformaldehyde for 3 hours, embedded in paraffin, and sectioned at 2 to 4 μm thickness. After deparaffinization, rehydration, and antigen retrieval in sodium citrate buffer, endogenous peroxidase activity was quenched with 3% hydrogen peroxide.^[18]^ Sections were then processed on a LEICA BOND-MAX fully automatic staining system. After incubation with primary antibodies, the sections were processed using the Bond Polymer Refine Detection kit (Leica Biosystems). This detection system utilizes a post-primary rabbit/mouse IgG linker followed by a Poly-HRP (horseradish peroxidase)-labeled polymer secondary antibody. This non-biotin, polymer-based method ensures high sensitivity and minimizes background interference from endogenous biotin. The enzymatic reaction was visualized using 3,3’-diaminobenzidine (DAB; part A: 2.4 mL, part B: 30 mL), and sections were counterstained with hematoxylin (30 mL). The primary antibodies for CELA1 (ab231120, clone EPR21004) and GZMK (67272-1-Ig, clone 1E11D1) were monoclonal. RBX1 (bs-8391R), AKR1C3 (11194-1-AP), and NDUFA4 (A09276-3) were polyclonal antibodies, which do not have specific clone designations. All antibodies were used according to the concentrations recommended by the manufacturers. Leica Application Suite was used for image acquisition. CELA1 staining was observed in cells morphologically consistent with fibroblasts and alveolar epithelial cells. NDUFA4 showed cytoplasmic staining in epithelial and stromal regions, whereas granzyme K staining was observed in infiltrating cells with lymphocyte-like morphology. RBX1 staining was observed mainly in tumor epithelial regions, and AKR1C3 staining was seen in bronchial epithelial and macrophage-like cells. Staining patterns were interpreted based on tissue morphology; subcellular localization and specific lymphocyte subsets were not further characterized. Immunohistochemical staining was assessed quantitatively using ImageJ, and the resulting image-analysis measurements were compared between benign and malignant nodules.

### 2.9. Statistical analysis

All analyses were conducted in R v4.1.4 (R Core Team). Continuous data are presented as mean ± SD or median (IQR) and compared by Student *t* test or Wilcoxon rank-sum test. Categorical variables are presented as n (%) and compared by χ^2^ or Fisher’s exact test. Differential expression was assessed with limma and FDR correction. LASSO modeling (Glmnet) used 10-fold CV via cv.glmnet, selecting minimum-error and 1-SE λ; this was repeated 3 times within an inner 5-fold loop. Selected genes entered a multivariate logistic regression (*P* < .05) to build the nomogram (rms). Calibration was evaluated by 1000-resample bootstrap (calibrate), discrimination by ROC/AUC with 95% CI (pROC) in outer 5-fold CV, and clinical utility by DCA (dca). Two-sided *P* < .05 denoted significance.

## 3. Results

### 3.1. MEgreen associated with malignant pulmonary nodules

To construct a scale-free co-expression network, we first analyzed the network topology for various soft-thresholding powers. As shown in Figure 1A, B, a soft threshold of β = 9 was selected to ensure the scale-free topology fit index (*R*^2^) reached the criterion of 0.8. Based on average hierarchical clustering and dynamic tree clipping, 9 distinct co-expression modules were identified (Fig. 1C). To identify the module most relevant to clinical status, we calculated the correlation between Module Eigengenes (MEs) – representing the overall expression levels of each module – and clinical traits. As illustrated in the module-trait relationship heatmap (Fig. 1D), the green module (MEgreen) exhibited the most significant positive correlation with malignant nodules (*r* = 0.57, *P* = 1 × 10^−9^).

**Figure 1.**
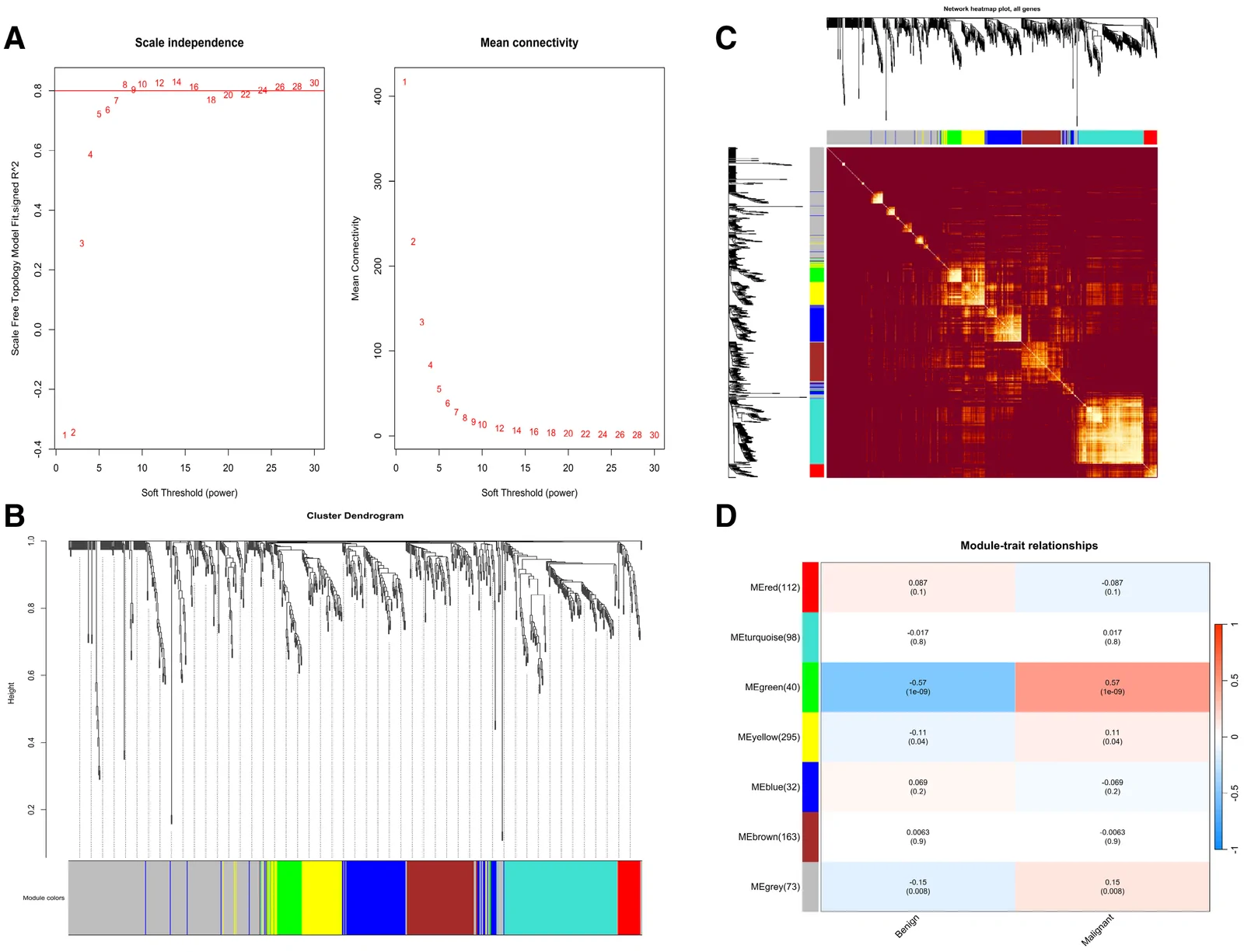
Visualization of WGCNA results. (A) Analysis of network topology for soft-thresholding powers. (B) A dendrogram of the clustered genes. (C) Topological overlap matrix heatmap. (D) Eigengene adjacency heatmap. (E) A heatmap showing the correlation between the gene module and clinical traits. WGCNA = weighted gene co-expression network analysis.

### 3.2. Functional enrichment analysis: GO and KEGG pathways

Enrichment analysis was performed for the brown, green, gray, red, and turquoise modules identified by WGCNA (Fig. 2), with particular attention to the green module because of its correlation with malignant nodule status. The green module was enriched for GO Molecular Function terms including oxidoreductase activity and Toll-like receptor binding (Fig. 2A). KEGG annotations included oxidative phosphorylation, prion disease, Parkinson disease, and nonalcoholic fatty liver disease (Fig. 2B). Enrichment profiles varied across modules, with NAD-related molecular functions observed in the brown module.

**Figure 2.**
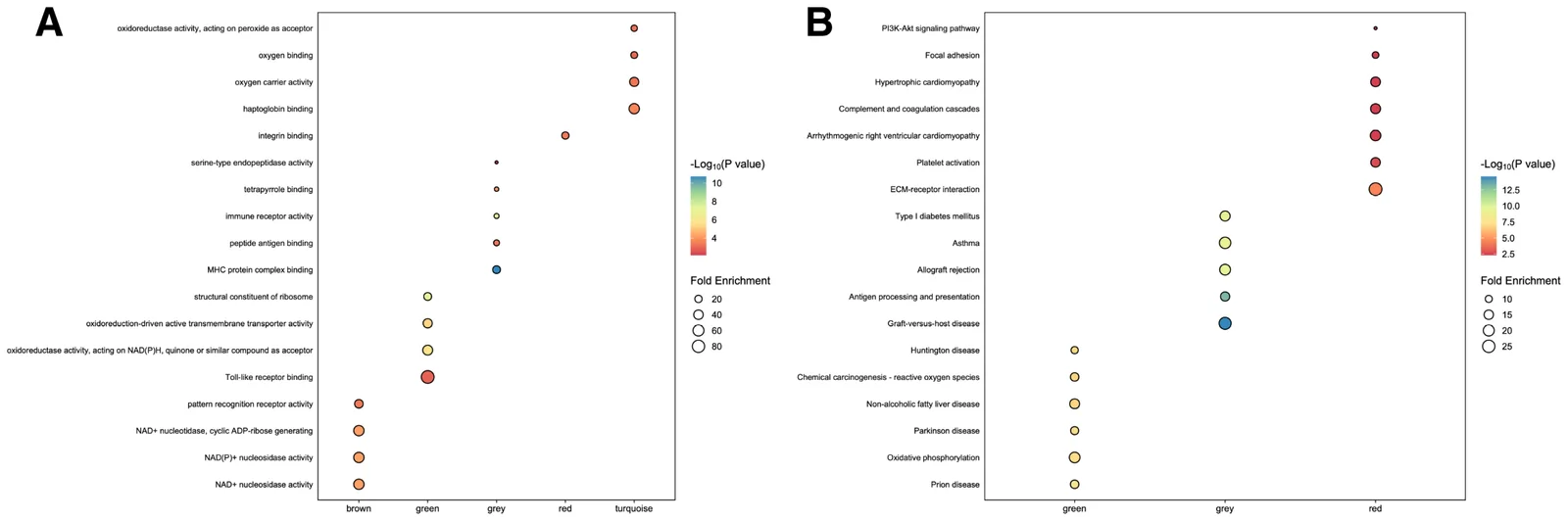
GO and KEGG enrichment analysis across co-expression modules. (A) GO Molecular Function annotations. (B) KEGG pathway annotations. The horizontal axis denotes the co-expression module, and the vertical axis lists the annotations. Bubble size represents fold enrichment, and color represents −log10(*P* value). GO = Gene ontology, KEGG = Kyoto Encyclopedia of Genes and Genomes.

### 3.3. Feature gene selection and function analysis

The intersection of differentially expressed genes and MEgreen module genes yielded 36 candidates (Fig. 3A). RPL9 and UQCRQ occupied central positions in the protein–protein interaction (PPI) network (Fig. 3B). Enrichment analysis identified annotations related to cellular stress responses, prion disease, and respiratory chain complex I (holoenzyme; Fig. 3C).

**Figure 3.**
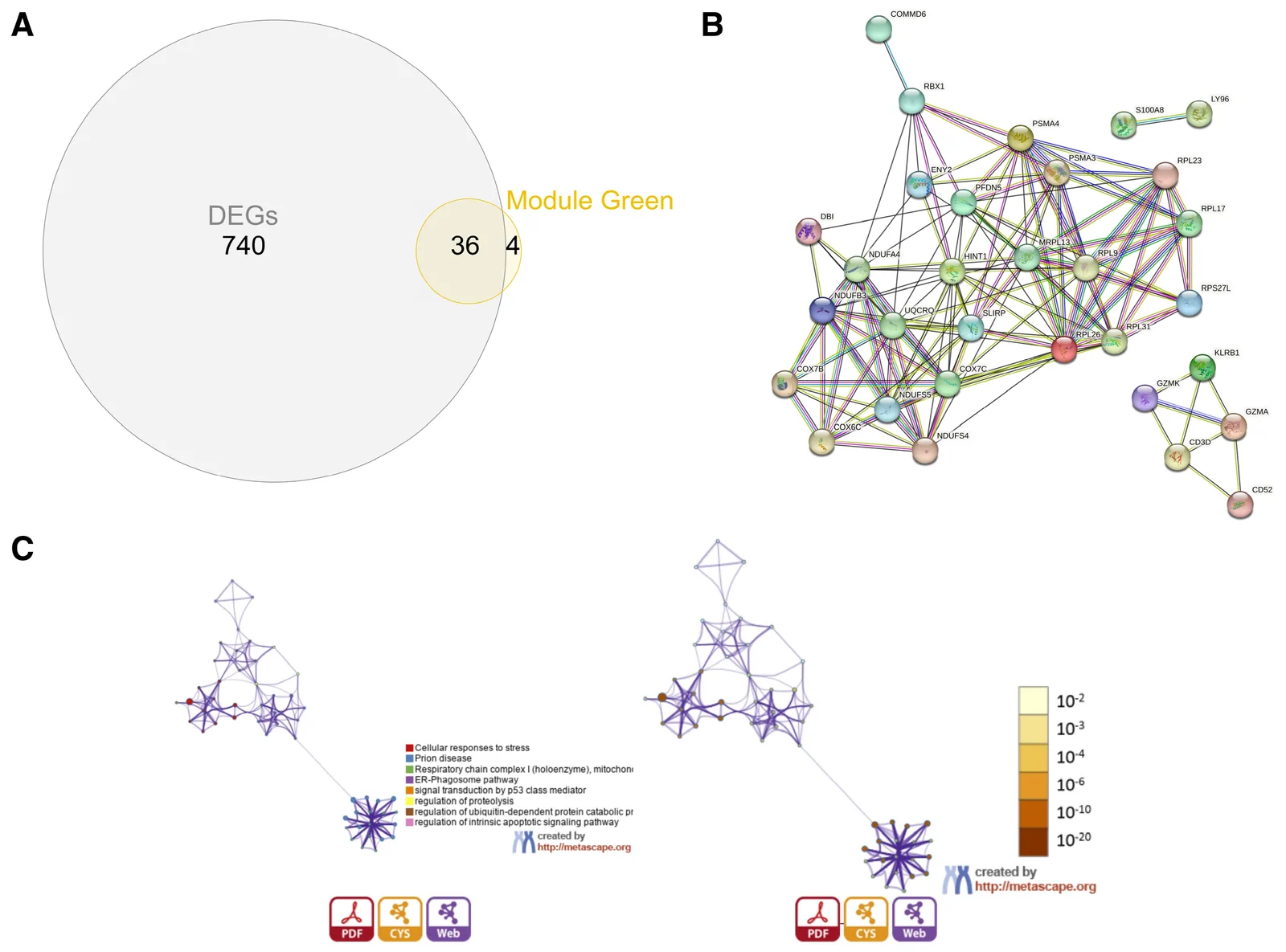
Feature gene selection. (A) Venn plot for intersecting genes between DEGs and genes in the MEgreen module. (B) Protein–protein interaction network. Each point represents a protein, and proteins are connected by lines. The number of lines represents the number of evidence chains. (C) Metascape function network. The redder the color, the more significant the enrichment result, and different colors represent different enrichment pathways. DEGs = differentially expressed genes.

### 3.4. Construction of the prediction model and performance assessment

Age, lesion size, and the 36 intersecting genes were considered as candidate predictors. LASSO selected age, lesion size, RBX1, GZMK, AKR1C3, NDUFA4, and CELA1 for model construction, with coefficients of 0.027, 0.052, 0.495, 0.273, 0.026, 0.111, and 0.421, respectively (*S* = 0.027 × Age + 0.052 × LesionSize + 0.495 × RBX1 + 0.273 × GZMK + 0.026 × AKR1C3 + 0.111 × NDUFA4 + 0.421 × CELA1) (as shown in Fig. 4A, B).

**Figure 4.**
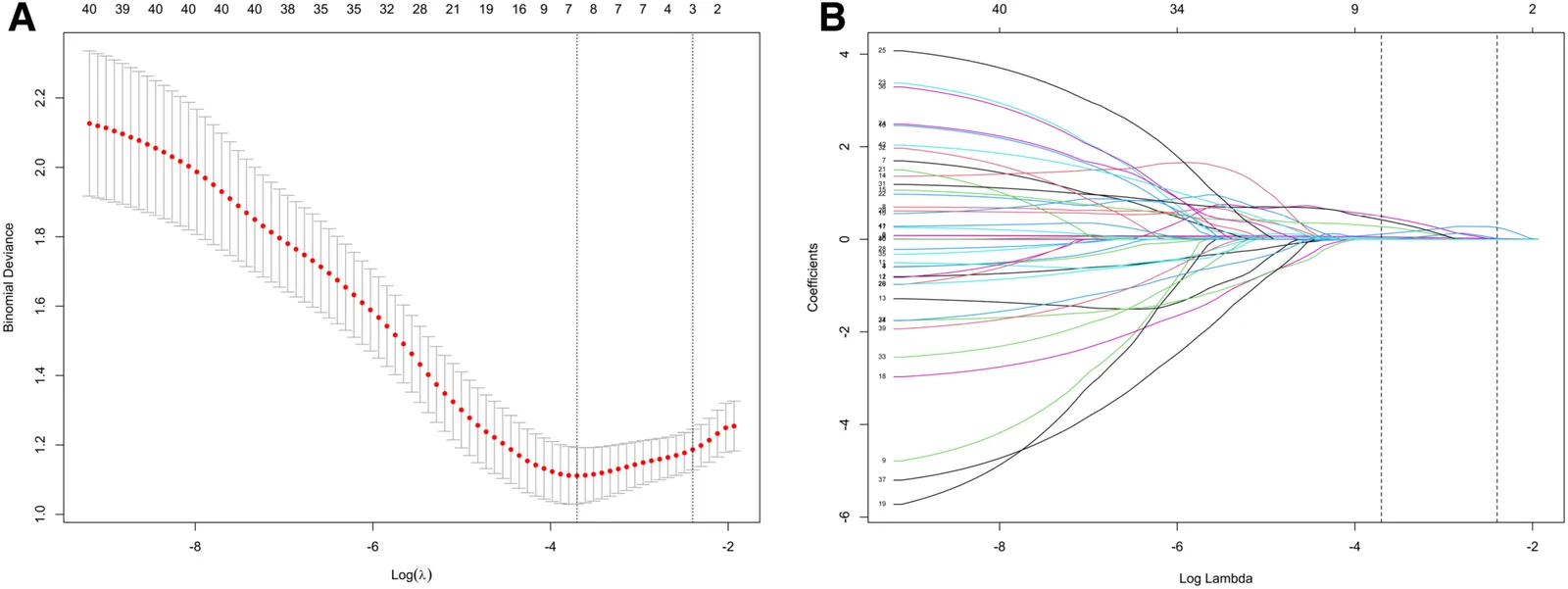
Feature gene selection based on LASSO regression. (A) Optimal lambda value selection. (B) Candidate gene selection based on optimal lambda. Each line represents a gene, and different genes are included based on different lambda values. The dashed line represents the genes that can be included at the optimal lambda value. LASSO = last absolute shrinkage and selection operator.

A nomogram was constructed using multivariate logistic regression (Fig. 5A). Age (OR = 1.05 [1.00, 1.11], *P* = .035 < .05) and lesion size (OR = 1.08 [95% CI: 1.04, 1.13], *P* < .001) were identified as risk factors for malignant nodules. However, the OR values for RBX1 (OR = 2.32 [95% CI: 0.55, 9.85], *P* = .252 > .05), GZMK (OR = 1.59 [95% CI: 0.82, 3.06], *P* = .168 > .05), AKR1C3 (OR = 1.07 [0.42, 2.72], *P* = .880 > .05), NDUFA4 (OR = 0.93 [0.24, 3.55], *P* = .911 > .05), and CELA1 (OR = 2.4 (0.69, 8.35), *P* = .169 > .05) did not show statistical significance.

**Figure 5.**
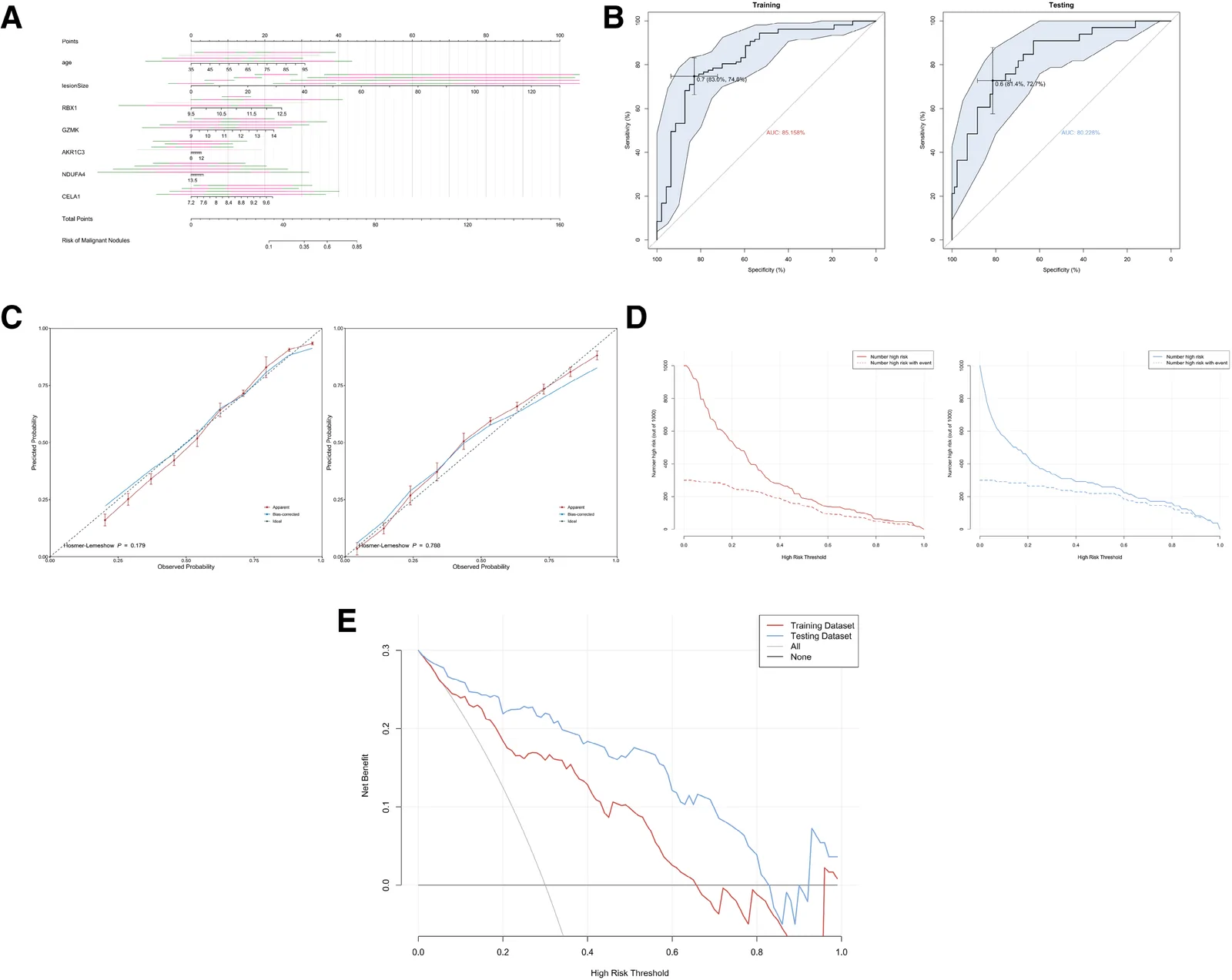
Construction and evaluation of the prediction model. (A) Nomogram of the predictive model. (B) Receiver operating characteristic (ROC) curves for the prediction model: left, training cohort; right, internal validation cohort. (C) Calibration plots for the prediction model: left, training cohort; right, internal validation cohort. (D) Clinical decision curve (CDC) analysis of the prediction model: left, training cohort; right, internal validation cohort.. (E) DCA curve of the prediction model in the validation set and test set. CDC = clinical decision curve, DCA = decision curve analysis, ROC = receiver operating characteristic.

In the training set, the AUC value was 85.16% (75.88–90.44%), while in the internal validation set it was 80.23% (70.08–89.38%; Fig. 5B and Table 2). The model was also evaluated in the external dataset (Fig. S1, Supplemental Digital Content 1, AUC = 0.867). The calibration of the prediction model was evaluated in both sets using calibration curves and the Hosmer–Lemeshow test. These curves demonstrated strong agreement between the predicted and actual malignancy of pulmonary nodules (Fig. 5C). Clinical impact curves (CIC) showed favorable alignment between the model-predicted and actual numbers of malignant nodules across probability thresholds (Fig. 5D). Decision curve analysis (DCA) indicated potential net benefit (Fig. 5E ).

**Table 2:** Parameter information of ROC curve in model validation.

	Training model	Internal validation
AUC	0.85234	0.80267
Sensitivity	0.815	0.78
Specificity	0.8025	0.7525
PPV	0.8046	0.7581
NPV	0.8132	0.7759

AUC = area under the curve, NPV = negative predictive value, PPV = positive predictive value, ROC = receiver operating characteristic curve.

### 3.5. GSVA analysis for prediction model

We computed pathway enrichment scores for benign and malignant pulmonary nodule samples through GSVA. Spearman correlation analysis was performed between pathway enrichment scores and model scores. In both the training and validation sets, the model score was found to be positively related to Notch, adherence junctions, non-small cell lung cancer, and the Wnt signaling pathway, and negatively related to ribosomes and the oxidative phosphorylation signaling pathway (Fig.6A, B).

**Figure 6.**
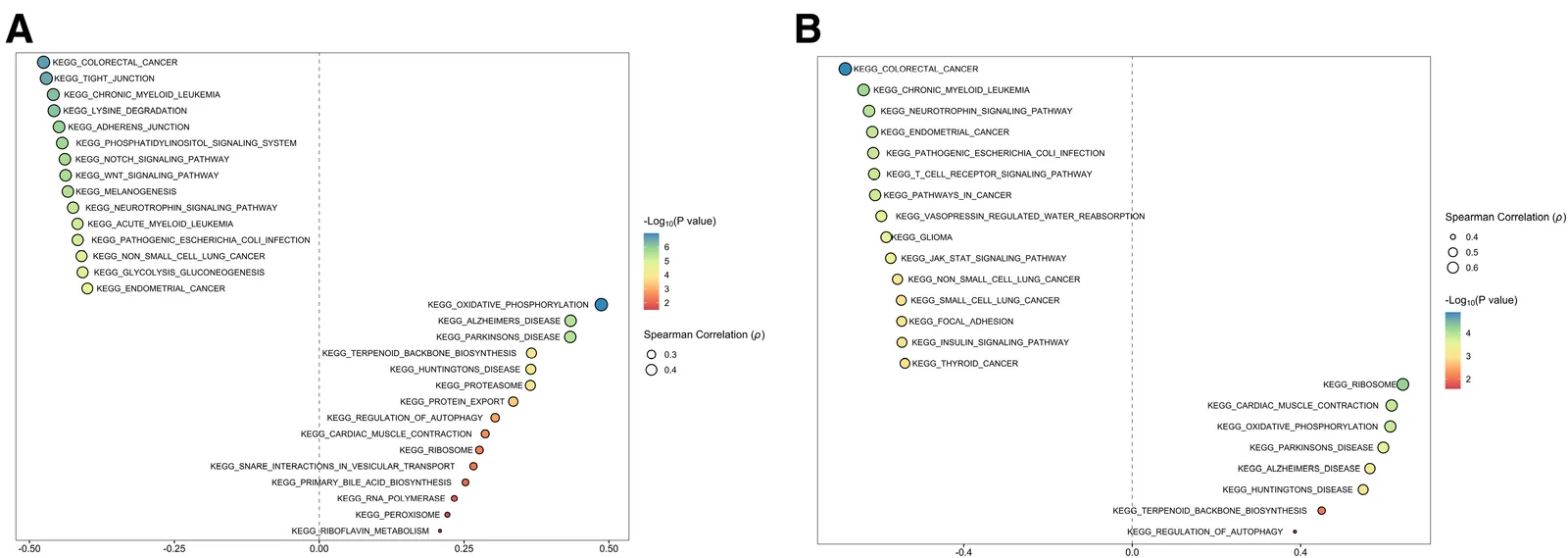
GSVA analysis of prediction model pathways. (A) Top 20 KEGG pathways from GSVA analysis, with bubble size indicating pathway contribution and color representing significance (red = more significant). (B) Top 20 KEGG pathways in an additional cohort, with similar sizing and coloring for contribution and significance. GSVA = gene set variation analysis, KEGG = Kyoto Encyclopedia of Genes and Genomes.

### 3.6. Validation of differential expression of model genes in a single-center clinical cohort

The single-center cohort included 15 patients with benign nodules and 15 with malignant nodules. The groups did not differ significantly in sex, age, smoking history, or maximum nodule diameter (Table 3). Blood RT-qPCR showed significantly higher CELA1 and NDUFA4 mRNA levels in the malignant group (Fig. 7A). In resected nodule tissue, immunohistochemistry (IHC) grayscale measurements for the corresponding proteins also differed significantly between groups (Fig. 7B). No significant group differences were observed for RBX1, GZMK, or AKR1C3 transcripts in blood or for their corresponding protein measurements in nodule tissue.

**Table 3:** Comparison of clinical information between 2 groups.

	Benign nodules (n = 15)	Malignant nodules (n = 15)	Sum (N = 30)	Statistic value	*P* Value
Age	54.47 ± 10.75	58 ± 9.91	56.23 ± 10.32	−0.936	.357
Gender				0	1
Female	9 (60%)	10 (66.67%)	19 (63.33%)		
Male	6 (40%)	5 (33.33%)	11 (36.67%)		
Smoking history				0	1
No	10 (66.67%)	11 (73.33%)	21 (70%)		
Yes	5 (33.33%)	4 (26.67%)	9 (30%)		
Maximum diameter	19.87 ± 7.27	16 ± 5.55	17.93 ± 6.65	1.637	.113

**Figure 7.**
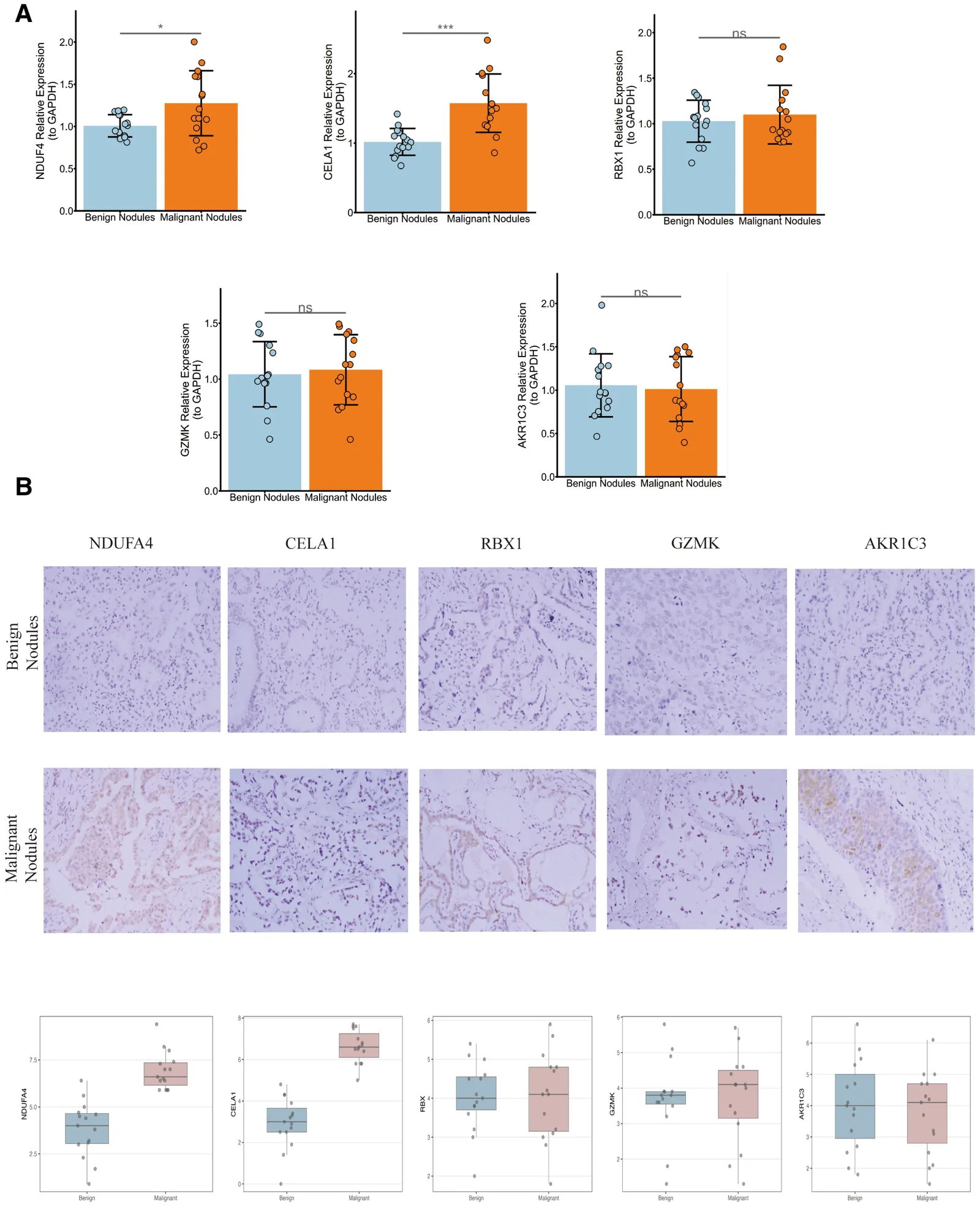
Blood mRNA expression and tissue IHC measurements in the single-center clinical cohort. (A) Relative mRNA expression of NDUFA4, CELA1, RBX1, GZMK, and AKR1C3 in blood, normalized to GAPDH. Bars represent means and error bars indicate standard deviations; dots represent individual sample values. (B) Representative IHC images and quantitative grayscale measurements obtained using ImageJ in benign and malignant nodule tissue. The GZMK label in panel B denotes granzyme K protein. Each group included 15 patients. IHC = immunohistochemistry, RBX1 = ring-box 1.

## 4. Discussion

Blood-based biomarkers have been investigated as adjuncts to clinical assessment of pulmonary nodules, including whole-blood transcriptomic classifiers,^[6]^ proteomic models,^[11]^ and models based on clinical factors.^[14]^ Our study combined 5 selected transcripts with age and lesion size, achieving AUCs of 85.16% and 80.23% in the training and internal validation cohorts, respectively. Candidate marker expression was also examined using blood RT-qPCR and nodule-tissue IHC in a single-center cohort. However, inflammatory diseases may also alter blood gene expression or blood cell composition and thereby affect model predictions. The present study did not separately assess the specificity of the 5-gene signature in patients with other inflammatory conditions. Validation in patients with nonmalignant inflammatory diseases is therefore needed to distinguish malignancy-associated expression patterns from those related to inflammation.

Early detection plays a crucial role in the global effort to combat lung cancer. Low-dose CT (LDCT) has been shown to reduce lung cancer mortality by 20% among smokers, defined as individuals older than 55 years with a smoking history exceeding 30 pack-years.^[17]^ However, the frequent bilateral and multifocal distribution of pulmonary nodules makes it impractical to perform biopsies on every CT-detected nodule. In patients with compromised pulmonary function or significant comorbidities, invasive biopsy may increase the risk of adverse outcomes.^[18,19]^ The detection of blood-based biomarkers offers a less invasive and more cost-effective strategy for discriminating between benign and malignant pulmonary nodules.^[9,20]^

By integrating whole-blood gene expression with age and lesion size, the model showed discrimination between benign and malignant nodules, with calibration analyses indicating agreement between predicted probabilities and observed outcomes. Decision curve analysis evaluated the potential value of using the combined gene-expression and clinical model to select patients for further diagnostic assessment. Net benefit balances correctly identifying malignant nodules against falsely classifying benign nodules as malignant, with the penalty for false-positive classifications determined by the threshold probability. The proposed application is model-assisted risk stratification alongside clinical assessment^[21]^; reductions in unnecessary investigations or improvements in patient outcomes were not directly evaluated.

The model development strategy combined WGCNA with differential expression analysis to identify candidate genes associated with nodule status, followed by LASSO and multivariable logistic regression to construct the prediction model. This approach links co-expression analysis with the selection of a limited set of predictors for an integrated nomogram.

RPL9 and UQCRQ occupied central positions in the PPI network, whereas LASSO selected RBX1, GZMK, AKR1C3, NDUFA4, and CELA1 for the prediction model. This difference reflects the distinct criteria used to characterize network connectivity and select predictors. The selected transcripts were combined with age and lesion size. Age was a risk factor for pulmonary nodules. Older patients have been consistently associated with a higher prevalence of malignant lesions.^[22]^ The relationship between age and lung cancer has also been clarified.^[23]^ The malignancy rate of pulmonary nodules increased significantly with age, with a malignant rate of <3% in people under 40 years old and approximately 50% in people over 60 years old.^[24,25]^ Lesion size also serves as a critical predictor. A large retrospective cohort study revealed that 85% of nodules larger than 10 mm in diameter were malignant,^[26]^ while 72% of the nodules smaller than 4 mm were benign.^[27]^ Sensitivity was larger than specificity when thresholds were set below 7 mm, while specificity was larger than sensitivity when thresholds were set above 7 mm.^[28]^

Ring-box 1 (RBX1) participates in ubiquitination through its interactions with cullin proteins. Previous tissue and experimental studies have associated RBX1 with NSCLC prognosis and lung tumorigenesis.^[29,30]^ In our clinical cohort, RBX1 transcript levels in blood and protein measurements in nodule tissue did not differ significantly between groups. NDUFA4 has been investigated in relation to cancer-cell proliferation and metabolism.^[31-35]^ Here, higher blood NDUFA4 transcript levels were accompanied by a difference in tissue IHC measurements. The relationship between these blood and tissue findings remains to be clarified.

Granzyme K, encoded by GZMK, has been studied in inflammatory and immune-cell contexts.^[36-43]^ In the present cohort, neither blood GZMK transcript levels nor tissue granzyme K measurements differed significantly between benign and malignant groups.

AKR1C3 has been investigated in several cancer settings.^[44-47]^ In our clinical cohort, AKR1C3 mRNA levels in blood and protein measurements in nodule tissue did not differ significantly between benign and malignant groups.

Chymotrypsin-like elastase 1 (CELA1) is involved in lung extracellular matrix remodeling.^[48]^ We observed higher CELA1 transcript levels in blood and a difference in CELA1 IHC measurements in nodule tissue from patients with malignant nodules. Together with the NDUFA4 findings, these observations support further investigation of the 2 markers, including their cellular sources and their relationship to nodule pathology.

The GSVA analysis examined the KEGG Notch signaling, Wnt signaling, adherens junction, non-small-cell lung cancer, ribosome, and oxidative phosphorylation gene sets. Previous work has examined Notch–EGFR interactions and stromal Notch signaling in lung cancer,^[49,50]^ Wnt signaling in lung branching,^[51]^ and epigenetic regulation in lung cancer.^[52,53]^ Studies of junctional signaling and epithelial–mesenchymal transition describe mechanisms regulating epithelial organization and changes in cell adhesion.^[54,55]^ In the present analysis, correlations between gene-set expression scores and model scores were measured in whole blood. Analyses of isolated peripheral blood mononuclear cells would be needed to determine whether the associations with these specific gene sets are present within that cell population.

Oxidative phosphorylation was enriched in the green module. Enrichment identifies an overrepresentation of genes assigned to a pathway, rather than measuring the direction or rate of pathway activity. Studies of tumor metabolism describe flexible use of glycolysis and oxidative phosphorylation,^[56]^ whereas lung studies examine respiratory-chain dysfunction and mitochondrial ribosomal stress in pulmonary pathology.^[56-58]^ These processes concern mitochondrial energy metabolism, but their activity in blood cells was not measured in this study. The enrichment of prion disease, Parkinson disease, and nonalcoholic fatty liver disease annotations may arise in part because mitochondrial respiratory-chain genes are included in several KEGG pathways. A respiratory-chain gene can contribute to both oxidative phosphorylation and disease-related annotations that incorporate mitochondrial dysfunction. Thus, the enrichment pattern may reflect genes associated with common mitochondrial processes rather than separate disease-specific mechanisms. This is a possible annotation-based explanation; the specific overlapping genes require verification against the enrichment results.

Several limitations should be considered. The experimental cohort was small and recruited at a single center, limiting the generalizability of the expression findings. Only CELA1 and NDUFA4 showed significant group differences in both blood transcript levels and tissue IHC measurements. The absence of significant differences for the other 3 markers may relate to sample size or between-patient heterogeneity and warrants evaluation in larger cohorts. Blood RT-qPCR and tissue IHC assessed different cell populations, and cell-specific and functional validation was not performed. In addition, family history and past medical history were unavailable for model development. Further studies should evaluate the 5-gene model in independent multicenter cohorts, including subgroups defined by nodule size, smoking history, and histological subtype, and assess its contribution to clinical management.

## 5. Conclusion

This study developed a prediction model integrating 5 blood transcriptomic markers with age and lesion size and evaluated its performance in internal and external datasets. CELA1 and NDUFA4 also showed group differences in blood transcript levels and nodule-tissue IHC measurements in a single-center cohort. These findings support further evaluation of the model for pulmonary nodule risk assessment and of the selected markers in cell-specific and functional studies.

## Author contributions

**Conceptualization:** Xingshu Zhang, Yongxiang Song

**Data curation:** Xingshu Zhang, Ming Gong

**Formal analysis:** Jie Yang, Ming Gong, Yongxiang Song

**Investigation:** Yongxiang Song

**Methodology:** Jie Yang, Qian Luo, Yongxiang Song

**Project administration:** Xingshu Zhang, Jie Yang, Ming Gong

**Resources:** Xingshu Zhang, Ming Gong, Qian Luo

**Software:** Xingshu Zhang, Jie Yang, Ming Gong, Qian Luo, Yongxiang Song

**Validation:** Xingshu Zhang, Jie Yang, Qian Luo, Yongxiang Song

**Visualization:** Jie Yang

**Writing – original draft:** Xingshu Zhang

**Writing – review & editing:** Xingshu Zhang, Jie Yang, Yongxiang Song


